# A targeted analysis identifies a high frequency of BRCA1 and BRCA2 mutation carriers in women with ovarian cancer from a founder population

**DOI:** 10.1186/s13048-015-0124-8

**Published:** 2015-03-27

**Authors:** Moria H Belanger, Lena Dolman, Suzanna L Arcand, Zhen Shen, George Chong, Anne-Marie Mes-Masson, Diane Provencher, Patricia N Tonin

**Affiliations:** Department of Human Genetics, McGill University, Montreal, Quebec Canada; The Research Institute of the McGill University Health Centre, Montreal, Quebec Canada; Molecular Pathology Centre, Department of Pathology, Jewish General Hospital, McGill University, Montreal, Quebec Canada; Département de médecine, Université de Montréal, Montreal, Canada; Centre de recherche du Centre Hospitalier de l’Université de Montréal and Institut du cancer de Montréal, Montreal, Quebec Canada; Division de gynécologie oncologique Université de Montréal, Montreal, Quebec Canada; Departments of Medicine, McGill University, Montreal, Quebec Canada; Department of Medical Genetics, Montreal General Hospital, Room L10-132, 1650 Cedar Avenue, Montreal, Quebec H3G 1A4 Canada

**Keywords:** BRCA1, BRCA2, Ovarian cancer, French Canadian, Mutation frequency, Genetic testing, Founder

## Abstract

**Background:**

The frequency of BRCA1 and BRCA2 mutations in ovarian cancer patients varies depending on histological subtype and population investigated. The six most commonly recurring BRCA1 and BRCA2 mutations previously identified in a founder French Canadian population were investigated in 439 histologically defined ovarian, fallopian tube and primary peritoneal cancer cases that were ascertained at one hospital servicing French Canadians. To further assess the frequency of BRCA1/BRCA2 mutations, a defined subgroup of 116 cases were investigated for all mutations previously reported in this population.

**Methods:**

A PCR-based assay was used to screen 439 ovarian, fallopian tube or extra-ovarian cancers comprised of serous, high grade endometrioid and mixed cell adenocarcinomas with serous components for specific BRCA1: C4446T and 2953delGTAinsC and BRCA2: 8765delAG, G6085T, 3398del5 and E3002K mutations. A multiplex bead-array-based Luminex assay was used to evaluate 19 specific mutations that have ever been reported in French Canadians, which included the six mutations assayed by PCR, in 116 cases representing all women ascertained within a defined 3-year window.

**Results:**

A targeted analysis of six mutations identified 34/439 (7.7%) mutation carriers and at least two mutation carriers for each mutation screened were found. The BRCA1:C4446T mutation was the most frequently identified variant (15/34, 44.1%) among mutation-positive cases. The expanded mutation screen that also included 13 additional variants identified 19/116 (16.4%) mutation carriers, where C4446T was the most common variant (8/19, 42.1%) identified among mutation-positive carriers in this subgroup. Mutations were identified in women with serous, endometrioid, mixed cell, and undifferentiated adenocarcinomas. Within this subgroup there were 73 high-grade (G3) serous ovarian carcinomas, the most common subtype, with mutations identified in 19.2% (n = 14) serous cases.

**Conclusions:**

Our results reaffirm that specific BRCA1 and BRCA2 mutations found previously to recur in French Canadian breast cancer and breast-ovarian cancer families, also recur in women with ovarian cancer not selected for family history of cancer. The high frequency of mutation carriers rationalizes genetic testing of ovarian cancer patients in this demographically defined population.

**Electronic supplementary material:**

The online version of this article (doi:10.1186/s13048-015-0124-8) contains supplementary material, which is available to authorized users.

## Background

Epithelial ovarian cancer is the fifth most frequent cause of cancer deaths in women in developed countries. The disease is heterogeneous, where serous, endometrioid, clear cell and mucinous carcinomas are the most common histological subtypes, and serous carcinomas, particularly high grade tumours, account for over 70% of all cases [1]. The poor overall survival rate at less than 30% is a reflection of the frequent diagnoses at advanced disease stage, high recurrence rates and development of resistance to chemotherapy [2,3]. Although there are no molecular predictors of outcome currently in clinical use [4], studies of specific cohorts suggest that hereditary genetic factors influence the outcome of ovarian cancer patients. Recently it has been observed that carriers of germline BRCA1 or BRCA2 mutations, which confer significantly increased risk for the hereditary form of ovarian cancer, exhibit higher response rates to platinum/paclitaxel chemotherapy (the standard of care for ovarian cancer), a longer progression-free survival, and improved overall survival as compared with non-carriers [5-7]. It has been proposed that defects in homologous recombination repair pathways affected by “Brca” dysfunction render the tumours sensitive to platinum-based DNA crosslinking agents and emerging therapies such as polyADP ribose (PARP) inhibitors [4,8]. Given these observations it has been recommended that identifying germline BRCA1/BRCA2 mutation carriers may have important implications in the management of women with ovarian cancer as targeted therapies such as PARP inhibitors are introduced into clinical settings [9].

The likelihood of harbouring a germline BRCA1/BRCA2 mutation depends on the histological subtype and/or tumour grade of epithelial ovarian cancers. Mutations are more frequently observed among women with high-grade serous or endometrioid subtype carcinomas as compared with low-grade serous/endometrioid, mucinous or clear cell subtype carcinomas [10,11]. Mutation carriers with undifferentiated adenocarcinomas of the ovary, as well as fallopian tube and primary peritoneal carcinomas, have also been identified [12-14]. Overall, BRCA1 and BRCA2 mutation carrier frequencies of 13-15% have been reported for ovarian cancer patients from the general population [11,12,15-17]. The high incidence of germline BRCA1 and BRCA2 mutations in ovarian cancer, has suggested that genetic assessment of women with ovarian cancer especially those with nonmucinous high-grade serous histology, will improve mutation carrier detection rates [14,18].

In order to assess the feasibility of offering genetic testing of women with ovarian cancer, it is important to determine the frequency of BRCA1 and BRCA2 mutation carriers in populations where genetic testing is available as the prevalence may vary. The large size and complex structure of each gene, the vast array of unique mutations identified, the spectrum of mutations, and the identification of variants of uncertain clinical significance has hampered genetic testing [19]. However, mutation detection has been facilitated by the prevalence of specific mutations identified in defined populations due to founder effects [19]. For example, significantly higher carrier frequencies (one in 40) have been reported for Ashkenazi Jewish women of Eastern European ancestry [20,21]. It is also well established that mutation carriers are significantly enriched in those women with ovarian cancer who also have a strong family history of breast and/or ovarian cancer, although a cancer family history is not a reliable method for identifying women with BRCA1/BRCA2 mutations [14,22,23].

French Canadian breast cancer and breast-ovarian cancer syndrome families of the province of Quebec harbour specific BRCA1 and BRCA2 mutations that recur in this demographically unique population due to founder effects attributed to common ancestors [24-32]. We have shown that five specific mutations (BRCA1: C4446T and 2953delGTAinsC and; BRCA2: 8765delAG, G6085T, 3398del5) account for the majority (85%) of all mutation-positive breast cancer and breast-ovarian cancer families, where index mutation carriers self-reported four grandparents of French Canadian descent [27] and haplotype analysis of these variants showed that they were likely due to common ancestors [25,26,29,33]. Although there are no official genetic testing guidelines in the province of Quebec, these research findings have informed genetic testing strategies (to reduce cost) in hereditary cancer clinics in Montreal, Quebec. Mutation testing of high risk French Canadians (self-reported ancestry of at least two or three grandparents), based on familial breast and ovarian cancer history, involves a screen for the most common variants and only when found negative is comprehensive screening performed by commercial testing by Myriad Genetics. A review of the BRCA1 and BRCA2 mutation spectrum found in French Canadian cancer families where comprehensive analysis was performed supports this strategy [10,24,28]. In Canada, all genetic testing for BRCA1 and BRCA2 must be ordered by a cancer genetics professional associated with a cancer medical genetics clinic, where clinics are managed by provincial health care jurisdictions. Although genetic testing for BRCA1 and BRCA2 for women with ovarian cancer is available in the neighbouring province of Ontario [34,35], referrals for genetic testing of ovarian cancer patients is on an *ad hoc* basis in Quebec.

In a targeted mutation analysis of BRCA1 (C4446T, 2953delGTAinsC, 3768insA) and BRCA2 (2816insA, 8765delAG, G6085T, 6503delTT) mutations found to recur at least twice in French Canadians, we reported a mutation carrier frequency of 8% in 99 women with epithelial ovarian cancer not selected for histological subtype or family history of cancer [36]. Mutation carriers were found among seven of 74 women with serous (N = 60) and endometrioid (n = 14) subtype carcinomas and one of four women with mixed cell subtype cancers, and none among the mucinous (n = 14) and clear cell (n = 7) subtype cancers [36]. Carriers harboured the BRCA1:C4446T, BRCA2:8765delAG and BRCA2:G6085T mutations, with C4446T being the most common mutation identified. These findings are in keeping with the carrier frequency of these variants in French Canadian breast cancer and breast-ovarian cancer families of the province of Quebec, Canada [24,27-29]. However, since our report on ovarian cancer, the spectrum of BRCA1 and BRCA2 mutations in this population has been further defined through comprehensive analyses of both genes, where 19 different pathogenic mutations in all have been found in French Canadian cancer families [24,26,27,37]. This includes the recurring BRCA2:3398del5 mutation found shared by common ancestors through haplotype analyses (Oros et al., [26]), and the recurring BRCA2:E3002K mutation, initially identified as variant of uncertain significance, and recently reclassified as a pathogenic mutation [37,38]. Thus the existing figure for the BRCA1 and BRCA2 mutation carrier frequency in French Canadian women with ovarian cancer may be an underestimate.

Due to the small sample size investigated in our initial study, we have assessed the frequency of the six most frequently reported mutations that were found to recur in French Canadian cancer families in a larger cohort of 439 (new) cases of histologically defined ovarian cancers that were from an ovarian cancer bank comprised of specimens collected from the largest French hospital system servicing gynecologic-oncology patients in Montreal, Quebec. This includes an evaluation of the newest recurring 3398del5 and E3002K mutations. To further assess the frequency of BRCA1 and BRCA2 mutations, a subgroup of 116 women with ovarian cancer ascertained within a three-year window were investigated for mutations using a broader panel of 19 mutations, which included the 13 additional variants previously reported in French Canadian cancer families and thus accounting for all BRCA1/BRCA2 mutations reported in French Canadians of Quebec. The findings from this study further define the spectrum and frequency of germline BRCA1/BRCA2 mutations in women with ovarian cancer not selected for family history of cancer and could further inform the development of mutation-screening policies for French Canadians in Quebec.

## Methods

### Study subjects

The study subjects were comprised of 439 women with epithelial ovarian (n = 429), fallopian tube (n = 7), or primary peritoneal (extra-ovarian) carcinomas (n = 3) that were collected as part of the ovarian tumour banking activities of the Banque de tissus et données of the Réseau de recherche sur le cancer of the Fond de recherche du Québec – Santé (Table 1). The tumour bank contains tumour tissues ascertained through surgeries that were collected at one hospital, the Centre hospitalier de l’Université de Montréal at the Notre Dame Hospital in Montreal, where at least 88% of cases self-reported French Canadian ancestry and over 98% consented to participate in banking activities. Samples from the tumour bank that were excluded from the study include: the women least likely to harbour germline BRCA1 or BRCA2 mutations, such as low-grade endometrioid, mucinous and clear cell carcinomas, or mixed cell cancers that did not have serous subtype components [10]; samples where DNA was no longer available for genetic testing; and samples banked prior to 1997 in order to avoid overlap with previously studied cases from our research group where BRCA1 and BRCA2 mutation frequencies had been investigated [36]. Clinical features (also collected as part of banking activities), such as disease stage, and tumour characteristics such as grade and histological subtype, were assigned by a gynaecologist-oncologist and gynecologic-pathologist, respectively, according to the criteria established by the International Federation of Gynecology and Obstetrics, and were provided by Système d’Archivage des Données en Oncologie (Table 1). Tumour grade noted as unclassified may have been a result of pre-operative exposure to chemotherapy, which has been known to affect histological subtype classification [39]. Disease stage was not available for one women with a serous ovarian carcinoma who was diagnosed at 91 years of age (Table 1).

**Table 1:** **Description of cases screened for BRCA1 and BRCA2 mutations**

**Group (Period of ascertainment, years)**	**Histological subtype** ^**1**^	**Number of women with ovarian cancer (fallopian tube** ^**2**^ **and primary peritoneal cancers)**	**Tumour grade**				**Disease stage** ^**3**^				**Mean age of diagnosis (range), years**
			**G1**	**G2**	**G3**	**Un-classified**	**I**	**II**	**III**	**IV**	
All (1997–2011)	Serous	369 (9)	12	62	279	16	25	28	275	40	61 (24 – 91)
	Endometrioid	30 (0)	0	20	9	1	13	2	14	1	58 (41 – 82)
	Mixed cell	32 (0)	0	11	21	0	6	2	21	3	57 (36 – 77)
	Undifferentiated	8 (1)	1^2^	0	3	4	2	0	6	0	61 (33 – 72)
	All types	439 (10)	13	93	312	21	46	32	316	44	61 (24 – 91)
Subgroup (2006–2008)	Serous	92 (4)	3	11	73	5	7	8	68	9	61 (38 – 82)
	Endometrioid	9 (0)	0	7	2	0	6	1	2	0	58 (41 – 82)
	Mixed cell	12 (0)	0	2	10	0	3	1	6	2	60 (40 – 77)
	Undifferentiated	3 (1)	1^2^	0	1	1	1	0	2	0	68 (62 – 71)
	All types	116 (5)	4	20	86	6	17	10	78	11	61 (38 – 82)

^1^Mixed cell has components of serous subtype cancer cells; ^2^fallopian tube adenocarcinoma; ^3^staging information was not available for a women with a serous ovarian carcinoma diagnosed at 91 years of age.

A personal history of cancer was noted for 51 women of whom 19 had synchronous cancers reported as follows: 29 were invasive breast cancer (three synchronous); ten endometrial cancers (all synchronous); four colorectal cancers (two synchronous); two cases of cervical cancer (one synchronous with a breast cancer); two lymphomas; and one case each of kidney cancer (synchronous), lung cancer (synchronous), multiple myeloma, retroperitoneal liposarcoma (synchronous) and skin cancer. Informed consent was obtained from all subjects at banking and, for this study, was performed in accordance with guidelines established by the institutional ethical review boards.

To further assess the frequency of BRCA1 and BRCA2 mutations, a subgroup of 116 women were selected for screening a broader panel of mutations previously reported in French Canadian cancer families as described below. Samples were selected from the 439 cases, and included all samples available according to our inclusion criteria that were ascertained from the period of 2006 to 2008 (Table 1).

### Mutation analysis

Mutation analysis was performed using DNA provided by the tissue bank that had been extracted from peripheral blood lymphocytes (n = 362) or fresh frozen tumour specimens (n = 77) when peripheral blood DNA was not available. All of the 439 women were evaluated for BRCA1 (C4446T, 2953delGTAinsC) and BRCA2 (8765delAG, G6085T, 3398del5, E3002K) mutations found to recur at least twice in breast and/or ovarian cancer families of French Canadian descent [24,26,27,29,37]. DNA samples were screened using established PCR-based screening assays [26,29] and/or by the Luminex platform as described below.

The Luminex platform was used to screen the subgroup of 116 cases (where 111 and five DNA samples were from peripheral blood lymphocytes or fresh frozen tumour specimens, respectively) for a broader panel of BRCA1 (1135insA, 2080insA, 2244insA, 2953delGTAinsC, 3768insA, 3875del4, 5221delTG, C4446T, E352X, G1081A, Q1846X) and BRCA2 (2816insA, 3034del4, 3398del5, 3773delTT, 6503delTT, 8765delAG, E3002K, G6085T) mutations, which included the six mutations screened in the whole cohort. Together this panel represented all pathogenic mutations identified in the French Canadians of Quebec that were known to us at the time of the study was conducted, and which included the most frequently recurring mutations screened by PCR-based assays [24,27,29,37]. The Luminex platform is a multiplex bead-array-based technology and was applied according to the manufacturer’s instructions with the xPONENT® software package for data management and analyses (www.luminexcorp.com).

The variants detected by either screening assay were verified by bidirectional Sanger Sequencing using 3730xl DNA Analyzer technology (Applied Biosystems) at the McGill University and Genome Quebec Innovation Centre (gqinnovationcenter.com). Sequencing chromatograms were analysed using Chromas^©^ Version 2.3 (Technelysium Pty Ltd) and compared with BRCA1 (U14680) or BRCA2 (U43746) GenBank reference sequences (www.ncbi.nlm.nih.gov/). The Human Genome Variation Society (HGVS) (www.hgvs.org) designation for each mutation is provided for all mutations investigated in this study. However, for historical reasons, the original mutation nomenclature is referred to throughout this article.

## Results

### The selected cases reflect the frequency and distribution of women with ovarian cancer identified in the general population

Table 1 describes the characteristics of the 439 women screened for the most recurrent BRCA1 and BRCA2 mutations identified in the French Canadian population and the subgroup of 116 cases screened for all of the mutations identified in the French Canadian population of Quebec. The subgroup represents all of the cases that were ascertained within a 2006–2008 window. We excluded from our analyses, low-grade endometrioid adenocarcinomas, mucinous cancers and mixed cell carcinomas lacking serous components, as these are least likely to harbour germline BRCA1/BRCA2 mutations [10,36]. The distribution of histological subtypes, tumour grade, disease stage and age at diagnosis within both groups are within the estimated frequencies observed for epithelial ovarian cancer subtypes found in the general population [10,36]. This is reflected in the observation that the majority of women (in the entire cohort and within the subgroup) are represented by high grade and late stage serous subtype ovarian carcinomas, with mean ages of diagnosis approximating 60 years of age.

### BRCA1:C4446T is the most frequently identified mutation in a screen of the most common recurring mutations in French Canadians

A previous study of French Canadian women with ovarian cancer was limited by both sample size investigated, and by spectrum of BRCA1 and BRCA2 mutations screened, where additional mutations have since been identified and found to recur in this population. [36]. Therefore to assess the contribution of recurring BRCA1/BRCA2 mutations in ovarian cancer cases from French Canadians, genomic DNA from 429 ovarian carcinomas and 10 fallopian tube or primary peritoneal carcinomas was screened for the presence of the two BRCA1 (C4446T, 2953delGTAinsC) and four BRCA2 (8765delAG, G6085T, 3398del5, E3002K) mutations using established PCR-based assays (Table 2). These specific mutations were selected for screening as they reflect the most commonly reported mutations found in French Canadian breast and/or ovarian cancer families based on the most recent observations from our group [24,26,27,29,37]. A targeted screening for these six specific mutations identified 34/439 (7.7%) mutation carriers, where at least two mutation carriers for each mutation screened were identified (Table 2). The BRCA1:C4446T mutation was the most frequently found variant (15/34, 44.1%) among mutation-positive cases. BRCA1 mutation-positive cases were found among the serous, endometrioid and mixed cell subtype adenocarcinomas in contrast to BRCA2 mutation carriers, which were exclusively found among the serous subtype tumours.

**Table 2:** **Frequency of the most common BRCA1 and BRCA2 mutations among French Canadian women with ovarian cancer**

**Histological subtype**	**Number of women with ovarian cancer (fallopian tube and primary peritoneal cancer)**	**BRCA1 positive (%)**			**BRCA2 positive (%)**					**BRCA1 and BRCA2 positive (%)**
		**C4446T**	**2953delGTAinsC**	**Total**	**8765delAG**	**G6085T**	**3398del5**	**E3002K**	**Total**	
Serous	369 (9)	12 (3.3)*	1 (0.3)	13 (3.5)	4 (1.1)	5 (1.4)*	3 (0.8)*	5 (1.4)*	17 (4.6)	30 (8.1)
Endometrioid	30 (0)	1 (3.3)	0	1 (3.3)	0	0	0	0	0	1 (3.3)
Mixed cell	32 (0)	2 (6.3)	1 (3.1)	3 (9.4)	0	0	0	0	0	3 (9.4)
Undifferentiated	8 (1)	0	0	0	0	0	0	0	0	0
All types	439 (10)	15 (3.4)	2 (0.5)	17 (3.9)	4 (0.9)	5 (1.1)	3 (0.7)	5 (1.1)	17 (3.9)	34 (7.7)

*One grade 2 mutation-positive carrier found among each of these mutation-positive carriers.

With one exception, that being a tumour sample harbouring a BRCA1:C4446T mutation, all identified mutations were detected in the germline. There was trace evidence for the normal allele in the sequencing chromatogram from the tumour sample containing the BRCA1 mutation. Although this BRCA1 mutation is the most commonly reported germline mutation in our cohort and in the French Canadian population, our finding does not exclude the possibility that this mutation was somatically derived.

### Genetic heterogeneity revealed by screening a broader panel of mutations in a defined subgroup of women with ovarian cancer

To further investigate the frequency and spectrum of BRCA1 and BRCA2 mutations in women with ovarian cancer, the analysis of a larger panel of mutations previously identified in French Canadian cancer families was performed using DNA samples from a subgroup of 116 of 439 women (Table 1). We focused our genetic analyses on this subgroup as it was not financially feasible to perform this assay on the entire cohort. We also selected this subgroup reasoning that it more accurately reflected a series. The screening panel was comprised of 11 BRCA1 and eight BRCA2 mutations (Table 3), which represented all of the BRCA1/BRCA2 mutations known to occur in French Canadians at the time the study was conducted, and included the six mutations that were analysed in the entire cohort of 439 cases (Table 2). All mutation-positive women who were initially identified by a targeted screen of six specific mutations using a PCR-based assay were also identified with the Luminex platform that was used to screen the subgroup of cases (data not shown).

**Table 3:** **Frequency of French Canadian BRCA1 and BRCA2 mutation carriers found in the defined subgroup of 116 women with cancer**

**Gene**	**Common mutation designation** ^**a**^	**HGVS mutation designation**	**Histological subtypes (%)**				
			**Serous (n = 92)**	**Endometrioid (n = 9)**	**Mixed cell (n = 12)**	**Undifferentiated (n = 3)**	**All types (n = 116)**
BRCA1	G1081A	c.962G > A	1 (1.1)	0	0	0	1 (0.9)
	1135insA	c.1016dupA	0	0	0	0	0
	E352X	c.1054G > T	0	1 (11.1)	0	0	1 (0.9)
	2080insA	c.1961dupA	0	0	0	0	0
	2244insA	c.2125_2126insA	1 (1.1)	0	1 (8.3)	0	2 (1.7)
	**2953delGTAinsC** ^**b**^	c.2834_2836delGTAinsC	0	0	1 (8.3)	0	1 (0.9)
	3768insA^b^	c.3649_3650insA	0	0	0	0	0
	3875del4	c.3756_3759delGTCT	0	0	0	0	0
	**C4446T** ^b^	c.4327C > T	8 (8.7)	0	0	0	8 (6.9)
	5221delTG	c.5102_5103delTG	0	0	0	0	0
	Q1846X	c.5536C > T	0	0	0	0	0
All BRCA1 variants			10 (10.9)	1 (11.1)	2 (16.7)	0	13 (11.2)
BRCA2	2816insA^b^	c.2588dupA	0	0	0	0	0
	3034del4	c.2806_2809delAAAC	0	0	0	0	0
	**3398del5**	c.3170_3174delAGAAA	0	0	0	0	0
	3773delTT	c.3545_3546delTT	0	0	0	1 (33.3)	1 (0.9)
	**G6085T** ^**b**^	c.5857G > T	1 (1.1)	0	0	0	1 (0.9)
	6503delTT^b^	c.6275_6276delTT	0	0	0	0	0
	**8765delAG** ^**b**^	c.8537_8538delAG	2 (2.2)	0	0	0	2 (1.7)
	**E3002K**	c.9004G > A	2^c^ (2.2)	0	0	0	2 (1.7)
All BRCA2 variants			5 (5.4)	0	0	1 (33.3)	6 (5.2)
All BRCA1 and BRCA2 variants			15 (16.3)	1 (11.1)	2 (16.7)	1 (33.3)	19 (16.4)

^a^The six mutations screened in the entire cohort of 439 women with cancer are bolded; ^b^Mutations screened in a previous assay of French Canadian women with ovarian cancer (Tonin et al., [36]). ^c^One mutation-positive carrier had a grade 2 carcinoma.

As shown in Table 3, this platform identified mutations in 19/116 (16.4%) women, where BRCA1:C4446T was the most common variant identified among mutation-positive cases (8/19, 42.1%) and examples of carriers of all six of the recurring mutations (see Table 2) were identified with the exception of BRCA2:3398del5 carriers. All mutations were found in the germline. The 19 mutation-positive carriers harboured one of nine different mutations identified from a total of 19 mutations screened by the Luminex platform (Table 3). Thus using the expanded screening panel 5/19 (26.3%) additional mutation-positive carriers were identified in this subgroup of 116 women, which included two carriers of the BRCA1:2244insA mutation. Examples of BRCA1 mutation carriers were found among women with serous, endometrioid and mixed cell subtype cancers in contrast to BRCA2 mutation carriers, who were found to have had either serous or undifferentiated adenocarcinomas.

### Comments on the clinical and tumour characteristics of mutation carriers

In this study, we identified a total of 39 mutation-positive carriers using either a PCR-based assay mutation screen for six specific mutations or multiplex screen of 19 mutations (Additional file 1: Table S1). For descriptive purposes, Table 4 summarizes disease stage and ages of diagnoses according to mutation status grouped by histotype. BRCA1 mutation-positive carriers were identified among all subtypes except for undifferentiated adenocarcinomas, whereas BRCA2 mutation-positive cases were identified among the serous and undifferentiated adenocarcinoma subtype cancers. Although the study was limited by screening for founder mutations only, there were no significant differences in distribution of BRCA1 and BRCA2 mutation-positive carriers based on comparisons of histological subtypes, tumour grade, and stage of disease (data not shown). Although mean age of cancer diagnosis of BRCA1 mutation carriers (53.8 years, range 36 – 76 years) was lower than BRCA2 mutation carriers (58.5 years, range 48 – 74 years) and women without mutations (61.3 years, range 24 – 91 years), this difference in age at diagnosis was not statistically significant, although this statistical analysis may have been limited by the small sample sizes for each comparison group.

**Table 4:** **Features of carriers based on BRCA1 and BRCA2 mutation status**

**Gene mutated**	**Histological subtype**	**Number of mutation carriers (n = 439) (%)**	**Age of diagnosis, years (%)**		**Tumour grade (%)**				**Disease stage** ^**1**^ **(%)**	
			**<60 (n = 196)**	**≥60 (n = 243)**	**G1 (n = 13)**	**G2 (n = 93)**	**G3 (n = 312)**	**Un-classified (n = 21)**	**I,II (n = 78)**	**III,IV (n = 360)**
BRCA1	Serous	15	9	6	0	1	14	0	3	12
	Endometrioid	2	2	0	0	0	2	0	0	2
	Mixed cell	4	3	1	0	0	4	0	0	4
	Undifferentiated	0	0	0	0	0	0	0	0	0
	All types	21	14	7	0	1	20	0	3	18
BRCA2	Serous	17	12	5	0	3	13	1	1	16
	Endometrioid	0	0	0	0	0	0	0	0	0
	Mixed cell	0	0	0	0	0	0	0	0	0
	Undifferentiated	1	0	1	0	0	1	0	0	1
	All types	18	12	6	0	3	14	1	1	17
BRCA1 or BRCA2	All types	39 (8.9)	26 (13.3)	13 (5.3)	0	4 (4.3)	34 (10.9)	1 (4.8)	4 (5.1)	35 (9.7)
None		400 (91.1)	170 (86.7)	230 (94.7)	13 (100)	89 (95.7)	278 (89.1)	20 (95.2)	74 (94.9)	325 (90.3)

^1^Staging information was not available for a women found mutation-negative who had a serous ovarian carcinoma diagnosed at 91 years of age.

There were 51 women that had a personal history of cancer that occurred prior to or occurred synchronously with an ovarian cancer diagnosis, with breast cancer (n = 29 cases) being the most common cancer reported (see Methods: study subjects section). Nine mutation carriers were identified in this group. One of these women with a prior history of cervical cancer diagnosed at 41 years of age was found to carry a BRCA1:C4446T mutation (Additional file 1: Table S1). The remaining eight mutation positive carriers were found among the women who had a prior diagnosis of breast cancer. Four of these women had breast cancer before age 51 years (Additional file 1: Table S1). Mutation carriers were found significantly among 27.6% (8/29) cases with a personal history of both breast and ovarian cancers as compared with women who did not (7.6% (31/410); p < 0.002).

## Discussion

Our study reaffirms that specific BRCA1 and BRCA2 mutations found previously to recur in French Canadian breast cancer and breast-ovarian cancer families, also recur in women with ovarian cancer not selected for family history of cancer. This is especially evident with the number of BRCA1:C4446T mutation carriers (n = 15) identified in this study, which has been the most commonly reported mutation identified in this population and this has been attributed to shared ancestry as a consequence of common founders [24,25,27-29,32,33]. This mutation was also the most common mutation found in our previous study of 74 women with serous and endometrioid ovarian cancers screened for specific BRCA1/BRCA2 mutations [36].

Our study also highlights the significance of the BRCA2:E3002K mutation in the French Canadian population. We found five E3002K mutation-positive carriers in the cohort of 439 women with ovarian cancer, which is similar in frequency to the number of carriers of each of the other three BRCA2 (8765delAG, G6085T, 3398del5) mutations in the same cohort. Although initially classified as a variant of uncertain clinical significance, both the recurrence of this mutation in breast and/or ovarian cancer families and results of an in vitro functional assay suggest that this variant is pathogenic [24,37,38].

A targeted screen of six specific mutations found to recur in French Canadian cancer families identified 7.7% of mutation-carrier cases among 439 women with ovarian, fallopian tube and primary peritoneal cancers. Examples of each mutation type were identified at least twice among mutation carriers, perhaps reflecting common founders in this population [25,29]. However, a high frequency of mutation carriers (16.4%) were identified in an analysis of a subgroup of 116 of the 439 women with cancer that were ascertained during a defined period of time using an expanded screen, which included the six most common mutations and an additional 13 mutations previously reported in French Canadian cancer families. The BRCA1:C4446T remains the most common mutation identified, accounting for 42.1% (8/19) of mutation-positive carriers, although the expanded screening panel identified an additional 26.3% (5/19) of mutation-positive carriers. This included the identification of two BRCA1:2244insA mutation carriers. This mutation has only been reported in one French Canadian cancer family in our studies [24,27]. During the course of this study, we further investigated the frequency of this mutation in the remaining cohort (n = 323) and found no other mutation-positives cases (data not shown). The results combined suggest that the population studied is more heterogeneous than previously suspected, and are consistent with recent analyses of French Canadian breast cancer and breast-ovarian cancer families where more comprehensive mutation methods were used [24].

From the genetic analysis of a subgroup of 116 women with ovarian cancer, we estimate a combined BRCA1 and BRCA2 mutation carrier frequency of 16.4% for ovarian, fallopian tube and primary peritoneal cancers classified as serous, high-grade endometrioid, mixed cell (containing a serous component) or undifferentiated adenocarcinomas not selected for family history of cancer. This estimate reflects carriers ascertained from the largest French hospital servicing gynecologic-oncology patients in Montreal. When matched only for the seven variants screened in common with our previous analysis (see Table 3), a mutation frequency of 10.1% (n = 12) was identified in the subgroup of 116 women with ovarian cancer as compared with our previous studies where 10.3% (n = 8) were identified in 78 histologically matched cases. The similarity in mutation frequencies is consistent with our earlier study suggesting the groups are similar. With respect to our previous study, expanding the mutation screen to include 12 other mutations captured an additional 5.9% (n = 7) of mutation-positive carriers. Within this subgroup there were 73 high-grade (G3) serous ovarian carcinomas, which represent the most common subtype of ovarian cancer, and mutations were identified in 19.2% (n = 14) cases. As our study was limited by the inability to perform a comprehensive screen of both BRCA1 and BRCA2 due to cost limitations and the use of banked samples rather than a series, the overall frequency of mutation carriers in ovarian cancer cases not selected for cancer family history remains unknown. The question thus remains whether BRCA1/BRCA2 mutations have been missed in the French Canadian population of Quebec. Notable however, is that our mutation screen of 19 specific mutations included all mutations that had been identified in French Canadian cancer families at the time the study was initiated. Indeed, targeted mutation screening analyses currently performed in hereditary cancer clinics of Montreal include either a subset or all of the 19 variants examined in our study in a targeted mutation screen of high-risk French Canadians. Thus although comprehensive (commercial) mutation screening follows a mutation-negative test, no new mutations have been introduced in targeted mutation screen for this population in hereditary cancer clinics of Montreal. Notable is that although our screen was targeted to 19 specific variants, our estimated carrier frequency of 16.4% is similar to the overall BRCA1 and BRCA2 mutation carrier frequency of 13-15% that has been reported for women with ovarian cancers not selected for family history or from demographically defined populations, where more comprehensive whole gene analyses were conducted [12,14-16,18].

Our carrier frequency in women with ovarian cancer is higher than a recently reported carrier frequency of 5.1% among French Canadian women with ductal carcinoma *in situ* or invasive breast cancer cases that were also ascertained from the same population [40]. However, only six of the 19 BRCA1 (C4446T, 2953delGTAinsC, and 3875del4) and BRCA2 (8765delAG, 3398del5, and G6085T) mutations screened in our study were evaluated in this independent report and thus it remains to be determined if the carrier frequency in ovarian cancer patients would exceed that found in women with breast cancer ascertained from the same population if similar genetic testing strategies are used.

Identifying carriers of BRCA1 and BRCA2 mutations allows for the targeted prevention of ovarian cancer in family members of ovarian cancer patients. As ovarian cancer surveillance has not proven effective, cancer prevention through salpingo-oophorectomy significantly reduces ovarian, fallopian and breast cancer risk in mutation carriers [41]. In Quebec, genetic testing is routinely offered to all women with a family history of breast cancer and breast-ovarian (epithelial type) cancer, and a personal history of breast and epithelial ovarian cancer through referral to hereditary cancer clinics. For women of French Canadian descent a targeted mutation analysis is performed based on the BRCA1 and BRCA2 mutations found to recur in this population as this is the most cost effective strategy for mutation detection given the high frequency of carriers harbouring founder mutations. Although mutation screening has been advocated for women with ovarian cancer in other jurisdictions in Canada, such as the province of Ontario [35], it is not currently routinely offered in Quebec. During the course of this investigation the Society for Gynecologic Oncology issued a clinical practice statement encouraging the medical community to offer genetic counselling and testing to all women with ovarian, fallopian tube and peritoneal carcinoma (www.sgo.org), but The Society of Gynecologic Oncology of Canada (www.g-o-c.org) has yet to propose its own recommendations.

An association with family history of breast and ovarian cancers cannot be commented upon in this study, as this information was not systematically ascertained with procurement of cases for tumour banking purposes. However, the apparent enrichment of BRCA1/BRCA2 mutation carriers among ovarian cancer cases with a prior or coincident invasive breast cancer would advocate genetic testing of ovarian cancer cases with breast cancer in the French Canadian population. Our previous study of ovarian cancer cases ascertained from the same population revealed that mutation status did not always correlate with a positive family history of breast and/or ovarian cancer [36] and this observation has also been made in independent studies of ovarian cancer [14] and more recently in French Canadian breast cancer cases not selected for family history of cancer [40]. Moreover, our previous findings that BRCA1 and BRCA2 mutations were enriched in serous, endometrioid and mixed cell histological subtype cancers from either unselected or familial cases [10,36], collectively would suggest that carrier detection could be advocated for French Canadian women who develop these subtypes of ovarian cancer, especially where cost of genetic testing remains an issue.

## Conclusions

Although our mutation analysis was targeted to identifying specific variants, mutation carriers were identified in 16.4% of cases in a defined subgroup of 116 cases, where 42.1% of mutation carriers harboured the most common BRCA1:C4446T mutation. Our results advocate for the expansion of the mutation panel used in clinical genetic testing for the French Canadian population of Quebec, possibly through use of a two-tier testing strategy (panel testing followed by full mutation screening if negative) as is currently available for French Canadian breast and ovarian cancer families in hereditary cancer clinics affiliated with McGill University and University of Montreal centres. Although the overall cost could be reduced for a defined population, population-based genetic testing has not been advocated due to the overall low frequency of carriers in the French Canadian population of Quebec [42]. As a significant number of ovarian cancer patients harbour germline BRCA1/BRCA2 mutations, a targeted screen for carriers in this defined population has the potential to improve health management of patients and reduce ovarian cancer risk for their mutation-carrier family members.

## Additional file

Additional file 1: Table S1. Characteristics of BRCA1 and BRCA2 mutation-positive carriers.

## References

[1] Seidman JD, Horkayne-Szakaly I, Haiba M, Boice CR, Kurman RJ, Ronnett BM (2004). The histologic type and stage distribution of ovarian carcinomas of surface epithelial origin. Int J Gynecol Pathol.

[2] NC I. Ovarian Epithelial Cancer Treatment (PDQ®). Bethesda, MA USA; 2012.

[3] Vaughan S, Coward JI, Bast RC, Berchuck A, Berek JS, Brenton JD (2011). Rethinking ovarian cancer: recommendations for improving outcomes. Nat Rev Cancer.

[4] Ledermann JA, Marth C, Carey MS, Birrer M, Bowtell DD, Kaye S (2011). Role of molecular agents and targeted therapy in clinical trials for women with ovarian cancer. Int J Gynecol Cancer.

[5] Yang D, Khan S, Sun Y, Hess K, Shmulevich I, Sood AK (2011). Association of BRCA1 and BRCA2 mutations with survival, chemotherapy sensitivity, and gene mutator phenotype in patients with ovarian cancer. JAMA.

[6] Vencken PM, Kriege M, Hoogwerf D, Beugelink S, van der Burg ME, Hooning MJ (2011). Chemosensitivity and outcome of BRCA1- and BRCA2-associated ovarian cancer patients after first-line chemotherapy compared with sporadic ovarian cancer patients. Ann Oncol.

[7] Hyman DM, Zhou Q, Iasonos A, Grisham RN, Arnold AG, Phillips MF (2012). Improved survival for BRCA2-associated serous ovarian cancer compared with both BRCA-negative and BRCA1-associated serous ovarian cancer. Cancer.

[8] Gelmon KA, Tischkowitz M, Mackay H, Swenerton K, Robidoux A, Tonkin K (2011). Olaparib in patients with recurrent high-grade serous or poorly differentiated ovarian carcinoma or triple-negative breast cancer: a phase 2, multicentre, open-label, non-randomised study. Lancet Oncol.

[9] Fong PC, Boss DS, Yap TA, Tutt A, Wu P, Mergui-Roelvink M (2009). Inhibition of poly(ADP-ribose) polymerase in tumors from BRCA mutation carriers. N Engl J Med.

[10] Tonin PN, Maugard CM, Perret C, Mes-Masson AM, Provencher DM (2007). A review of histopathological subtypes of ovarian cancer in BRCA-related French Canadian cancer families. Fam Cancer.

[11] Pal T, Permuth-Wey J, Betts JA, Krischer JP, Fiorica J, Arango H (2005). BRCA1 and BRCA2 mutations account for a large proportion of ovarian carcinoma cases. Cancer.

[12] Alsop K, Fereday S, Meldrum C, deFazio A, Emmanuel C, George J (2012). BRCA mutation frequency and patterns of treatment response in BRCA mutation-positive women with ovarian cancer: a report from the Australian Ovarian Cancer Study Group. J Clin Oncol.

[13] Vicus D, Finch A, Cass I, Rosen B, Murphy J, Fan I (2010). Prevalence of BRCA1 and BRCA2 germ line mutations among women with carcinoma of the fallopian tube. Gynecol Oncol.

[14] Walsh T, Casadei S, Lee MK, Pennil CC, Nord AS, Thornton AM (2011). Mutations in 12 genes for inherited ovarian, fallopian tube, and peritoneal carcinoma identified by massively parallel sequencing. Proc Natl Acad Sci U S A.

[15] Zhang S, Royer R, Li S, McLaughlin JR, Rosen B, Risch HA (2011). Frequencies of BRCA1 and BRCA2 mutations among 1,342 unselected patients with invasive ovarian cancer. Gynecol Oncol.

[16] McLaughlin JR, Rosen B, Moody J, Pal T, Fan I, Shaw PA (2013). Long-term ovarian cancer survival associated with mutation in BRCA1 or BRCA2. J Natl Cancer Inst.

[17] Kurian AW (2010). BRCA1 and BRCA2 mutations across race and ethnicity: distribution and clinical implications. Curr Opin Obstet Gynecol.

[18] Schrader KA, Hurlburt J, Kalloger SE, Hansford S, Young S, Huntsman DG (2012). Germline BRCA1 and BRCA2 mutations in ovarian cancer: utility of a histology-based referral strategy. Obstet Gynecol.

[19] Szabo C, Masiello A, Ryan JF, Brody LC (2000). The breast cancer information core: database design, structure, and scope. Hum Mutat.

[20] Oddoux CSJ, Clayton CM, Neuhausen S, Brody LC, Kaback M, Haas B (1996). The carrier frequency of the BRCA2 6174delT mutation among Ashkenazi Jewish individuals is approximately 1%. Nat Genet.

[21] Roa BB, Boyd AA, Volcik K, Richards CS (1996). Ashkenazi Jewish population frequencies for common mutations in BRCA1 and BRCA2. Nat Genet.

[22] Tonin PN, Perret C, Lambert JA, Paradis AJ, Kantemiroff T, Benoit MH (2001). Founder BRCA1 and BRCA2 mutations in early-onset French Canadian breast cancer cases unselected for family history. Int J Cancer.

[23] King MCMJ, Mandell JB, New York Breast Cancer Study Group (2003). Breast and ovarian cancer risks due to inherited mutations in BRCA1 and BRCA2. Science.

[24] Cavallone L, Arcand SL, Maugard CM, Nolet S, Gaboury LA, Mes-Masson AM (2010). Comprehensive BRCA1 and BRCA2 mutation analyses and review of French Canadian families with at least three cases of breast cancer. Fam Cancer.

[25] Manning AP, Abelovich D, Ghadirian P, Lambert JA, Frappier D, Provencher D (2001). Haplotype analysis of BRCA2 8765delAG mutation carriers in French Canadian and Yemenite Jewish hereditary breast cancer families. Hum Hered.

[26] Oros KK, Leblanc G, Arcand SL, Shen Z, Perret C, Mes-Masson AM (2006). Haplotype analysis suggest common founders in carriers of the recurrent BRCA2 mutation, 3398delAAAAG, in French Canadian hereditary breast and/ovarian cancer families. BMC Med Genet.

[27] Oros KKGP, Greenwood CM, Perret C, Shen Z, Paredes Y, Arcand SL (2004). Significant proportion of breast and/or ovarian cancer families of French Canadian descent harbor 1 of 5 BRCA1 and BRCA2 mutations. Int J Cancer.

[28] Simard J, Dumont M, Moisan AM, Gaborieau V, Malouin H, Durocher F (2007). Evaluation of BRCA1 and BRCA2 mutation prevalence, risk prediction models and a multistep testing approach in French-Canadian families with high risk of breast and ovarian cancer. J Med Genet.

[29] Tonin PN, Mes-Masson AM, Futreal PA, Morgan K, Mahon M, Foulkes WD (1998). Founder BRCA1 and BRCA2 mutations in French Canadian breast and ovarian cancer families. Am J Hum Genet.

[30] Scriver CR (2001). Human genetics: lessons from Quebec populations. Annu Rev Genomics Hum Genet..

[31] Laberge AM, Michaud J, Richter A, Lemyre E, Lambert M, Brais B (2005). Population history and its impact on medical genetics in Quebec. Clin Genet.

[32] Tonin PN (2006). The limited spectrum of pathogenic BRCA1 and BRCA2 mutations in the French Canadian breast and breast-ovarian cancer families, a founder population of Quebec. Canada. Bull Cancer..

[33] Vezina H, Durocher F, Dumont M, Houde L, Szabo C, Tranchant M (2005). Molecular and genealogical characterization of the R1443X BRCA1 mutation in high-risk French-Canadian breast/ovarian cancer families. Hum Genet.

[34] Metcalfe KA, Fan I, McLaughlin J, Risch HA, Rosen B, Murphy J (2009). Uptake of clinical genetic testing for ovarian cancer in Ontario: a population-based study. Gynecol Oncol.

[35] Finch A, Bacopulos S, Rosen B, Fan I, Bradley L, Risch H (2014). Preventing ovarian cancer through genetic testing: a population-based study. Clin Genet.

[36] Tonin PN, Mes-Masson AM, Narod SA, Ghadirian P, Provencher D (1999). Founder BRCA1 and BRCA2 mutations in French Canadian ovarian cancer cases unselected for family history. Clin Genet.

[37] Cote S, Arcand SL, Royer R, Nolet S, Mes-Masson AM, Ghadirian P (2012). The BRCA2 c.9004G > A (E2002K) [corrected] variant is likely pathogenic and recurs in breast and/or ovarian cancer families of French Canadian descent. Breast Cancer Res Treat.

[38] Guidugli L, Pankratz VS, Singh N, Thompson J, Erding CA, Engel C (2013). A classification model for BRCA2 DNA binding domain missense variants based on homology-directed repair activity. Cancer Res.

[39] McCluggage WG, Lyness RW, Atkinson RJ, Dobbs SP, Harley I, McClelland HR (2002). Morphological effects of chemotherapy on ovarian carcinoma. J Clin Pathol.

[40] Ghadirian P, Robidoux A, Nassif E, Martin G, Potvin C, Patocskai E (2014). Screening for BRCA1 and BRCA2 mutations among French-Canadian breast cancer cases attending an outpatient clinic in Montreal. Clin Genet.

[41] Rebbeck TR, Kauff ND, Domchek SM (2009). Meta-analysis of risk reduction estimates associated with risk-reducing salpingo-oophorectomy in BRCA1 or BRCA2 mutation carriers. J Natl Cancer Inst.

[42] Ghadirian P, Robidoux A, Zhang P, Royer R, Akbari M, Zhang S (2009). The contribution of founder mutations to early-onset breast cancer in French-Canadian women. Clin Genet.

