# Supplementary material for: A targeted analysis identifies a high frequency of BRCA1 and BRCA2 mutation carriers in women with ovarian cancer from a founder population
Source: J Ovarian Res. 2015 Mar 27;8:1. doi: 10.1186/s13048-015-0124-8 (PMC4376165; doi:10.1186/s13048-015-0124-8)
Supplement: Additional file 1: Table S1. — Characteristics of BRCA1 and BRCA2 mutation-positive carriers. [file 13048_2015_124_MOESM1_ESM.docx]

| Additional file 1: Table S1. Characteristics of BRCA1 and BRCA2 mutation-positive carriers | | | | | | | |
| --- | --- | --- | --- | --- | --- | --- | --- |
| Sample ID^1^ | Gene | Mutation^2^ | Histological subtype | Grade | Disease stage | Age of diagnosis (years) | Personal history of other cancers (age at diagnosis, years) |
| **2658** | BRCA1 | 2244insA | Mixed cell: serous and clear cell | G3 | IV | 47 |  |
| **4010** | BRCA1 | 2244insA | Papillary serous cystadenocarcinoma | G3 | IIB | 52 | Breast cancer 51 |
| 820 | BRCA1 | 2953delGTA+C | Papillary serous cystadenocarcinoma | G3 | IIIC | 72 | Breast cancer 65 |
| **3360** | BRCA1 | 2953delGTA+C | Mixed cell: serous and endometrioid | G3 | IIIC | 49 |  |
| 747 | BRCA1 | C4446T | Serous cystadenocarcinoma | G3 | IIIC | 60 | Breast cancer 55 |
| 893 | BRCA1 | C4446T | Endometrioid adenocarcinoma | G3 | IV | 57 |  |
| 951 | BRCA1 | C4446T | Serous cystadenocarcinoma | G2 | IC | 46 |  |
| 1151 | BRCA1 | C4446T | Mixed cell: serous and endometrioid | G3 | IIIC | 46 |  |
| 1793 | BRCA1 | C4446T | Serous cystadenocarcinoma | G3 | IIIC | 54 |  |
| **2194** | BRCA1 | C4446T | Papillary serous cystadenocarcinoma | G3 | IC | 43 | Breast cancer 40 |
| 2627 | BRCA1 | C4446T | Serous cystadenocarcinoma | G3 | IIIC | 36 |  |
| **2885** | BRCA1 | C4446T | Papillary serous cystadenocarcinoma | G3 | IIIC | 53 |  |
| **3510** | BRCA1 | C4446T | Papillary serous cystadenocarcinoma | G3 | IIIC | 60 |  |
| **3621** | BRCA1 | C4446T | Serous cystadenocarcinoma | G3 | IIIC | 64 |  |
| **3844** | BRCA1 | C4446T | Serous cystadenocarcinoma | G3 | IIIC | 51 |  |
| **3853** | BRCA1 | C4446T | Serous cystadenocarcinoma | G3 | IIIC | 76 |  |
| **3872** | BRCA1 | C4446T | Papillary serous cystadenocarcinoma | G3 | IIIC | 63 | Breast cancer 49 |
| **4057** | BRCA1 | C4446T | Papillary serous cystadenocarcinoma | G3 | IIIC | 40 | Breast cancer 31 |
| 2069 | BRCA1 | C4446T | Mixed cell: serous and mucinous | G3 | IIIC | 63 |  |
| **3205** | BRCA1 | E352X | Endometrioid adenocarcinoma | G3 | IIIC | 49 |  |
| **3929** | BRCA1 | G1081A | Serous cystadenocarcinoma | G3 | IV | 49 | Cervical cancer 41 |
| 4447 | BRCA2 | 3398del5 | Papillary serous cystadenocarcinoma | G3 | IIIC | 64 |  |
| 5205 | BRCA2 | 3398del5 | Papillary serous cystadenocarcinoma | G2 | IIIC | 59 |  |
| 5312 | BRCA2 | 3398del5 | Serous cystadenocarcinoma | G3 | IV | 53 |  |
| **4330** | BRCA2 | 3773delTT | Undifferentiated adenocarcinoma | G3 | IIIC | 71 |  |
| 867 | BRCA2 | 8765delAG | Papillary serous cystadenocarcinoma | G3 | IIIC | 55 |  |
| 1144 | BRCA2 | 8765delAG | Papillary serous cystadenocarcinoma | G3 | IIIC | 56 |  |
| **2912** | BRCA2 | 8765delAG | Papillary serous cystadenocarcinoma | G3 | IIIC | 53 |  |
| **4104** | BRCA2 | 8765delAG | Serous cystadenocarcinoma | G3 | IIIC | 59 |  |
| 721 | BRCA2 | E3002K | Serous cystadenocarcinoma | G3 | IV | 74 |  |
| 794 | BRCA2 | E3002K | Papillary serous cystadenocarcinoma | G3 | IIIC | 58 |  |
| **3029** | BRCA2 | E3002K | Papillary serous cystadenocarcinoma | G2 | IIIC | 74 |  |
| **3698** | BRCA2 | E3002K | Serous cystadenocarcinoma | G3 | IIIC | 53 | Breast cancer 49 |
| 5238 | BRCA2 | E3002K | Serous cystadenocarcinoma | G3 | IV | 62 |  |
| 1136 | BRCA2 | G6085T | Serous cystadenocarcinoma | G3 | IIIC | 59 |  |
| 1272 | BRCA2 | G6085T | Papillary serous cystadenocarcinoma | G2 | IIIC | 50 |  |
| 2557 | BRCA2 | G6085T | Papillary serous cystadenocarcinoma | G3 | IIIC | 48 |  |
| **4323** | BRCA2 | G6085T | Papillary serous cystadenocarcinoma | G3 | IIC | 48 |  |
| 4453 | BRCA2 | G6085T | Serous cystadenocarcinoma | Unclassified | IIIC | 70 | Breast cancer 65 |
| ^1^Samples identification (ID) in bold are cases screened by the Luminex platform; ^2^Common BRCA1/BRCA2 mutation nomenclature (HGSV designation listed in Table 3) | | | | | | | |
